# Early warning and risk assessment of cardiac involvement in dermatomyositis/polymyositis: from biomarkers to multimodal imaging

**DOI:** 10.3389/fmed.2026.1987083

**Published:** 2026-10-21

**Authors:** Wenqing Liu, Yuping Jiang, Shu Li, Chenxu Song, Kaihua Liu, Manman Hou, Luping Zhao, Wenhao Li, Bin Yan, Sen Mao

**Affiliations:** 1 College of Medical Imaging and Laboratory, Jining Medical University, Jining, China; 2 Department of Ultrasound Medicine, the Affiliated Hospital of Jining Medical University, Jining, China; 3 Department of Rehabilitation Medicine, the Affiliated Hospital of Jining Medical University, Jining, China; 4 Department of Medical Imaging, the Affiliated Hospital of Jining Medical University, Jining, China

**Keywords:** biomarkers, cardiac involvement, dermatomyositis, multimodal imaging, polymyositis

## Abstract

Idiopathic inflammatory myopathies (IIM) comprise a broad spectrum of clinical and pathological subtypes, including dermatomyositis (DM), anti-synthetase syndrome, immune-mediated necrotising myopathy (IMNM), overlap myositis, inclusion-body myositis, and polymyositis (PM). Among these, DM remains well-defined, whereas PM has become increasingly rare under current classification criteria. A significant number of early studies categorised patients collectively under the term “DM/PM”. Cardiac involvement is recognised as a significant cause of mortality in these patients. The reported incidence of cardiac involvement in DM/PM varies widely and is frequently subclinical. The symptoms are frequently masked by manifestations in the skeletal muscles and the skin, which makes an early diagnosis difficult using conventional examination methods. Although endomyocardial biopsy is regarded as the gold standard, its invasive nature limits its clinical application. It is therefore imperative to establish an efficient framework for early warning and precise risk assessment of cardiac involvement in DM/PM. In this review, we summarize current and emerging evidence on serum biomarkers and multimodal imaging for the early identification and risk assessment of cardiac involvement in DM/PM. These advances are built on the evolving classification system for IIM.The integration of these tools with clinical high-risk factors is intended to facilitate the construction of a comprehensive risk assessment model. This integrated approach is intended to facilitate the early detection and treatment of cardiac involvement in DM/PM patients, thereby improving their prognosis.

## 1 Introduction

Idiopathic Inflammatory Myopathies (IIM) include subtypes such as dermatomyositis (DM), antisynthetase syndrome, immune-mediated necrotizing myopathy(IMNM), overlap myositis, inclusion body myositis, and polymyositis (PM). As a subtype of IIM, DM is distinguished by the presence of distinctive cutaneous lesions and systemic symptoms that manifest in a variety of clinical forms (1). PM also belongs to the IIM subgroup, with pathological features of lymphocytic infiltration surrounding non-necrotic endomysial muscle fibers (2). Despite the fact that PM has become a rare diagnosis under the current clinicopathological and serological classification criteria, and many patients previously diagnosed with PM have been reclassified into other IIM subtypes, we maintain the use of this terminology when discussing prior studies. This observation aligns with the findings reported in numerous earlier literatures, which have documented cardiac involvement in combined DM/PM cohorts. Notwithstanding the discrepancies in clinical presentations, both DM and PM are characterized by symmetrical proximal muscle weakness (3). The fundamental pathological change that characterizes this condition is the presence of inflammatory infiltration of striated muscle fibers. This phenomenon can affect not only skeletal muscle and skin but also the myocardium, which is another type of striated muscle. The cardiac manifestations associated with this condition are diverse, and may include heart failure, myocarditis, and cardiac conduction abnormalities (4). Clinically overt cardiac involvement is relatively uncommon in patients with DM/PM; nevertheless, cardiovascular involvement remains an important cause of death.

The reported incidence of cardiac involvement in patients with DM/PM varies widely in the literature, ranging from 6% to 72% (5, 6). Among these, approximately 32.5% to 72% of patients exhibit subclinical cardiac abnormalities characterized by electrocardiographic changes (7, 8). These alterations occur in the absence of overt clinical symptoms and may be obscured by manifestations of skeletal muscle disease and cutaneous involvement. Consequently, a significant proportion of patients already exhibit irreversible structural and functional cardiac injury at the time of diagnosis, and this insidious presentation poses substantial challenges for clinical identification and management. Despite the fact that endomyocardial biopsy remains the gold standard for the diagnosis of cardiomyopathy (9), its invasive nature restricts its widespread clinical use. Consequently, the identification of efficient and specific early warning markers, in conjunction with the establishment of a precise risk assessment framework, is of considerable clinical significance for improving patient outcomes, by enabling earlier detection and intervention for cardiac involvement in DM/PM. Biomarkers offer the advantages of quantitative assessment and ease of measurement, allowing more accurate reflection of early pathological processes and serving as valuable tools for early disease detection. Concurrently, multimodal imaging techniques provide direct visualization of cardiac architecture, function, and histopathological changes, thereby constituting a reliable basis for precise evaluation of the morphological and functional manifestations of cardiac involvement. Our article summarizes recent advances in biomarkers and imaging studies and discusses strategies for early warning and risk assessment of cardiac involvement in patients with DM/PM, with the aim of informing clinical practice.

## 2 Cardiac involvement in DM/PM

Although extramuscular manifestations are common in DM/PM (4), the pathological essence of inflammatory infiltration of striated muscle fibers renders the myocardium susceptible to damage, with diverse manifestations of involvement. A meta-analysis by Xiong A et al. (10), encompassing 22 studies, demonstrated that patients with DM/PM exhibit a significantly elevated risk of cardiovascular events in comparison to the general population [relative risk [RR] = 2.37, 95% confidence interval [CI]: 1.86–3.02]. This elevated risk was associated with both PM (RR = 2.35, 95% CI: 1.51–3.66) and DM (RR = 2.55, 95% CI: 1.66–3.93). Subgroup analysis revealed a significantly increased risk of ischemic heart disease (RR = 1.76, 95% CI: 1.40–2.21), indicating that DM/PM may be an independent risk factor for the development of ischemic heart disease. Cardiac involvement in DM/PM is a common cause of death, typically manifesting as heart failure, arrhythmias, and myocardial infarction (4). Heart failure is the most frequently reported cardiac manifestation clinically, with an incidence ranging from 32% to 77% (11). In a study by Yafasova A et al. (12), 936 patients with DM/PM were compared to 3,744 matched non-DM/PM controls. The 10-year absolute risk for a new-onset heart failure outcome was found to be 6.98% (CI: 5.16%–9.16%) in DM/PM patients, versus 4.58% (CI: 3.79%–5.47%) in matched controls (*P* = 0.002). For atrial fibrillation, the corresponding risk was 10.17% (CI: 7.94–12.71%) versus 7.07% (CI: 6.09%–8.15%) in the control group (*P* = 0.005). The study concluded that patients with DM/PM have a higher associated risk of developing heart failure and other adverse cardiac outcomes, as well as higher mortality, compared with matched controls.

The interpretation of epidemiological studies on cardiovascular outcomes in DM/PM requires caution. Although several studies used age- and sex-matched control populations, such matching alone may not adequately control for important confounders. Traditional cardiovascular risk factors, including hypertension, diabetes mellitus, dyslipidemia, obesity, smoking, and older age, may independently increase the risk of heart failure, arrhythmia, ischemic events, and overall cardiovascular morbidity. In addition, treatment-related factors, particularly cumulative glucocorticoid exposure, may contribute substantially to adverse cardiovascular outcomes through metabolic and vascular effects. Therefore, in studies where these variables were not comprehensively assessed or adjusted for, the observed excess cardiovascular risk should not be attributed solely to direct myocardial involvement caused by IIM. Rather, it is likely to reflect an interaction between disease-specific inflammatory injury, treatment exposure, and background cardiovascular risk burden.

In the majority of patients diagnosed with DM/PM, cardiac abnormalities are present but remain subclinical (4). Although electrocardiographic changes are the most common manifestations, the true prevalence of known electrocardiographic abnormalities compared to age- and sex-matched populations remains unclear due to a paucity of controlled trials. Consequently, routine electrocardiography and echocardiography frequently prove inadequate for the early and accurate diagnosis of heart conditions. In order to facilitate timely intervention, it is of the utmost importance to identify high-risk individuals at an early stage, to explore pathways for early warning signs, and to conduct appropriate risk assessments. A number of risk factors have been identified in research studies as being associated with cardiac involvement in DM/PM. Multivariate analyses indicate that long disease duration, high global disease activity scores, and the presence of interstitial lung disease (13, 14) are independent risk factors for cardiac damage in these patients. Furthermore, traditional cardiovascular risk factors are more prevalent in patients with DM/PM and may act synergistically with systemic inflammation to accelerate cardiac pathology (15).

## 3 Early warning with biomarkers for cardiac involvement in DM/PM

### 3.1 Traditional biomarkers of myocardial injury

The injury and necrosis of cardiomyocytes are primarily reflected by traditional myocardial injury biomarkers. Clinically, these biomarkers can be used for diagnosing myocardial damage and also serve as fundamental early-warning indicators for cardiac involvement in DM/PM.

Serum cardiac troponin (cTn) is a biomarker frequently employed in the diagnosis of myocardial injury, exhibiting high sensitivity and cardiac specificity. This characteristic facilitates the detection of myocardial damage at an early stage (16–18). Troponin comprises two forms: cardiac troponin I (cTnI) and cardiac troponin T (cTnT). cTnI is released exclusively from cardiomyocytes (19). It functions as a pivotal regulatory element in the processes of myocardial contraction and relaxation (20), inducing conformational changes in its target proteins. In a study by Lilleker JB et al. (21) investigating different myocardial injury biomarkers for identifying cardiac involvement in IIM, cardiac involvement was present in 62% of IIM patients with abnormal cTnI, compared to only 21% in those with abnormal cTnT. Consequently, cTnI can be regarded as having superior specificity and positive predictive value in comparison to cTnT (22) and is a reliable indicator for the assessment of subclinical myocardial involvement in DM/PM. In contrast to cTnI, cTnT can also be derived from adult skeletal muscle (19, 22, 23). Consequently, elevated cTnT levels in patients with DM/PM may be indicative of skeletal muscle injury, which can be indistinguishable from myocardial damage. In clinical practice, this can be differentiated by combining it with other indicators, or by using cTnI in conjunction to improve the accuracy of determining the presence of cardiac involvement in DM/PM patients.

Creatine kinase (CK) is one of the enzymes released following cardiomyocyte injury and is commonly used in the diagnosis of myocardial infarction (24), serving as a reliable indicator of myocardial damage. Research by Hak AE et al. (25) found that approximately 20% to 90% of patients with DM/PM exhibit elevated CK levels, ranging from 2 to 10 times the normal range. Patients presenting with symptoms of muscle weakness are observed to have elevated CK levels with greater frequency. For individual patients already in remission, an increase in serum CK activity may predict disease relapse. Furthermore, Borleffs JC et al. (26) noted in a prospective study that, although preliminary reports suggested changes in serum myoglobin levels might precede CK changes in DM/PM patients, CK holds greater value for these patients, who often have chronic muscle damage, whereas myoglobin is more sensitive to acute muscle injury (Figure 1).

**Figure 1:**
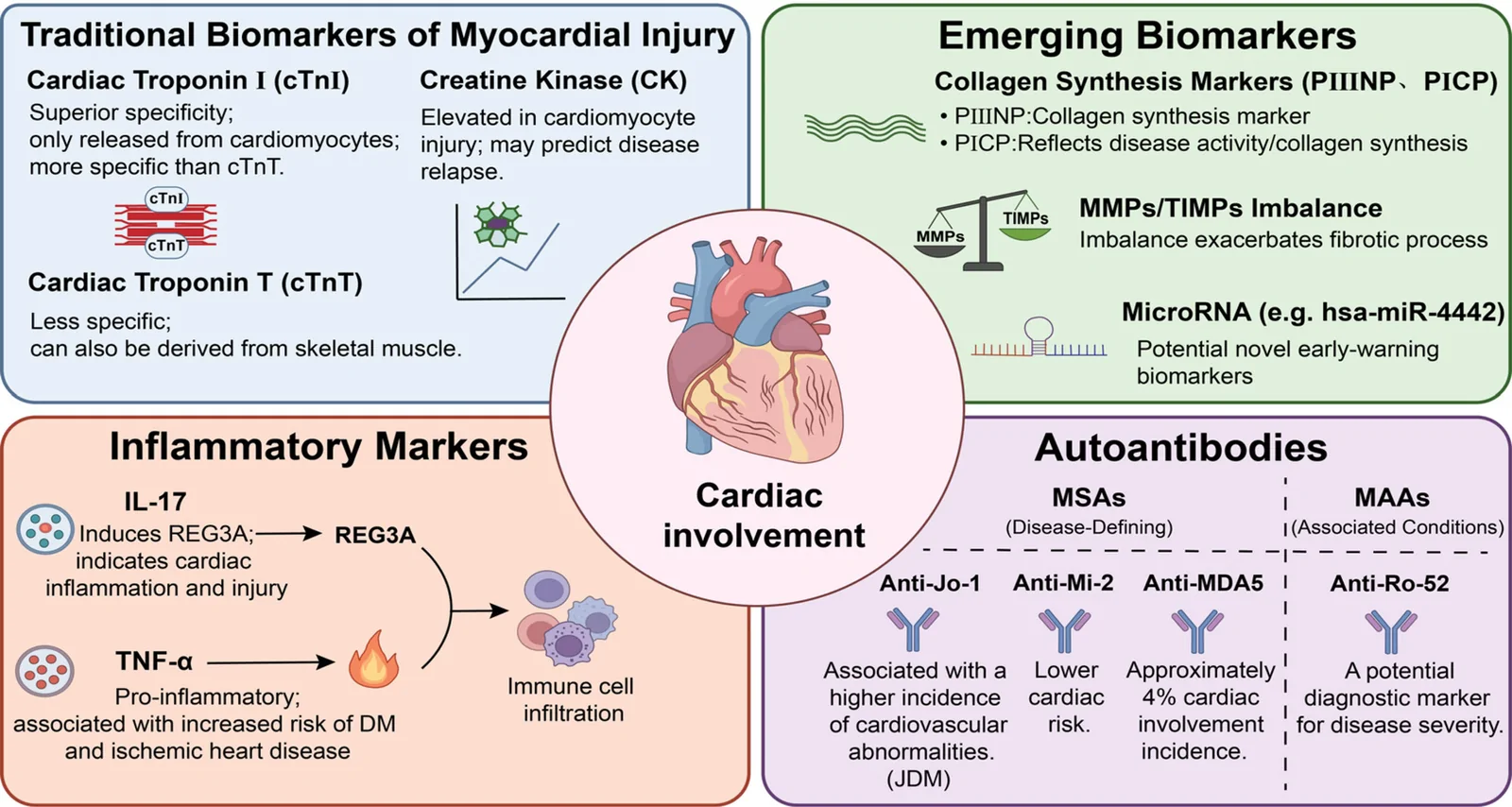
Early warning with biomarkers for cardiac involvement in DM/PM. Four key categories are depicted: Traditional biomarkers of myocardial injury, emerging biomarkers, inflammatory markers and autoantibodies.

### 3.2 Emerging biomarkers

The advancement of molecular biology techniques has resulted in the continuous discovery of emerging biomarkers with greater predictive value. These emerging biomarkers have the capacity to compensate for the limitations of traditional markers, offering higher sensitivity and specificity.

Myocardial fibrosis is one of the crucial pathological alterations in cardiac involvement of DM/PM, which can lead to irreversible cardiac damage. A substantial body of evidence suggests that the procollagen Ⅲ N-terminal peptide (PⅢNP) is the most frequently and extensively studied biomarker of myocardial fibrosis (27, 28), reflecting the active rate of collagen synthesis. Kubo M et al. (29) demonstrated that the serum level of the carboxyterminal propeptide of type I procollagen (PⅠCP) functions as an indicator of active collagen synthesis in patients with various fibrotic diseases. The study, which measured serum PICP levels in 20 DM patients and 29 controls, found that the mean PICP level in DM patients was significantly higher than that in the normal control group (mean ± SD: 326 ± 76 ng/mL vs 135 ± 88 ng/mL; *P* < 0.001), with elevated serum PICP levels observed in 60% of the DM patients. This finding suggests that serum PICP levels may serve as a diagnostic tool for detecting disease activity or severity in DM. In summary, PⅢNP and PICP may serve as early warning indicators for the onset of myocardial fibrosis in DM/PM, thereby helping to avert irreversible cardiac injury and providing a basis for clinical intervention. Matrix metalloproteinases (MMPs) have been shown to degrade myocardial collagen, while their inhibitors, tissue inhibitors of metalloproteinases (TIMPs), have been demonstrated to suppress MMPs activity. It is evident that both of these enzymes play a crucial role in the regulation of myocardial fibrosis, and an imbalance between them has been observed to exacerbate the fibrotic process. Kieseier BC et al. (30) found that mRNA expression levels of MMP-2 and MMP-9 were significantly elevated in DM/PM patients, whereas TIMPs expression levels showed no significant change compared to controls. Immunohistochemical results demonstrated that MMP-1 was primarily localized around the sarcolemma of affected muscle fibers and fibroblast-like cells, while MMP-9 was mainly expressed by infiltrating T lymphocytes, suggesting its pivotal role in inflammatory muscle tissue. The present study indicates that MMPs play a pathogenic role in the development and progression of inflammatory myopathies. Consequently, it can be deduced that alterations in MMPs may serve as a reflection of the dynamic process of myocardial fibrosis in DM/PM patients with cardiac involvement, thereby unveiling novel prospects for the early warning and disease monitoring of cardiac complications.

MicroRNAs (miRNAs) are a class of non-coding small RNA molecules that have been shown to play a crucial role in regulating key biological processes. They have emerged as a promising source of novel early-warning biomarkers for cardiac involvement in DM/PM. In the study by Hirai T et al. (31), the plasma levels of miRNAs were analyzed in patients with both DM/PM and other active autoimmune diseases, as well as in healthy controls. The analysis was conducted before and after treatment. The results showed that the plasma levels of hsa-miR-4442 were significantly higher in patients with active DM/PM than in patients with other active autoimmune diseases or healthy controls. This finding suggests that plasma levels of miRNAs may be associated with DM/PM, and hsa-miR-4442 has been reported to be elevated in active DM/PM; however, current evidence mainly reflects overall disease activity rather than cardiac-specific involvement. Therefore, it should be regarded as an exploratory biomarker, and its value in early identification of myocardial involvement remains unproven. Subsequent research could delve deeper into its expression profile in DM/PM patients with cardiac involvement, investigate whether its changes correlate with the severity of myocardial inflammation and fibrosis and track its dynamic trends. This would provide supplementary evidence for cardiac risk assessment and, by expanding the study sample size, facilitate the translation of research findings into clinical applications (Figure 1).

### 3.3 Inflammatory markers

The fundamental mechanism of cardiac involvement in DM/PM is attributed to autoimmunity-mediated mononuclear inflammatory cell infiltration (4). Inflammatory markers have been shown to reflect the inflammatory status and disease activity of patients, thereby serving as an early warning system for the severity of cardiac inflammation. Interleukin (IL) is one of the pivotal cytokines involved in the autoimmune inflammatory response of DM/PM, mediating inflammatory infiltration and injury of cardiomyocytes. Furthermore, IL-17 has been demonstrated to induce a significant elevation of Regenerating Islet-derived Protein 3*α* (REG3A) in DM/PM (32). It has been demonstrated through rigorous research that REG3A has the capacity to serve as an indicator of the extent of cardiac inflammation and injury (33). Therefore, IL-17 could be considered a potential biomarker for cardiac involvement in DM/PM, providing a basis for early warning. Tumor Necrosis Factor-α (TNF-α) is a potent pro-inflammatory cytokine that plays a significant role in inflammation and immune responses. A meta-analysis evaluating the association between the TNF-α-308A/G polymorphism and the risk of DM indicated that this polymorphism might be a potential risk factor for DM susceptibility (34). A further meta-analysis indicated that the TNF-α-308A allele may be associated with an elevated risk of ischemic heart disease in the general population (35). Consequently, elevated serum TNF-α levels in DM/PM patients may serve as an auxiliary warning indicator, indicating an increased risk of cardiac involvement (Figure 1).

### 3.4 Autoantibodies

As autoimmune diseases, a core pathological feature of DM/PM is the presence of autoantibodies. These autoantibodies are categorized into two distinct groups: myositis-specific autoantibodies (MSAs) and myositis-associated autoantibodies (MAAs) (36). MSAs are unique to DM/PM and are closely associated with disease subtypes and organ involvement. Classic MSAs include anti-Jo-1 antibody, anti-Mi-2 antibody, and anti-MDA5 antibody, among others, while anti-Ro52 antibody is one of the most common MAAs.

The presence of the anti-Jo-1 antibody has been detected in approximately 15%–25% of patients diagnosed with DM/PM, constituting the most prevalent anti-aminoacyl-tRNA synthetase (anti-ARS) antibody identified to date (37). Despite the paucity of reports associating the anti-Jo-1 antibody with cardiac involvement in DM/PM, a study focusing on juvenile dermatomyositis (JDM) (38) has indicated a higher incidence of cardiovascular abnormalities in patients who are positive for this antibody. The anti-Mi-2 antibody has been demonstrated to be significantly associated with both DM and PM, showing a stronger correlation with DM than with PM (39). Patients who are positive for this antibody often present with characteristic skin lesions, while the incidence of cardiac involvement is relatively low. However, a negative anti-Mi-2 antibody result does not exclude the possibility of cardiac involvement, necessitating assessment in conjunction with other indicators. Patients with anti-MDA5 antibody-positive DM frequently exhibit rapidly progressive interstitial lung disease and also carry a risk of cardiac involvement. As reported by Wu W. et al. (40), the incidence of cardiac involvement (e.g., new-onset atrial fibrillation or elevated cardiac troponin) in subject with MDA5^+^ DM is approximately 4%. Xu L. et al. (41) conducted a retrospective study of 265 anti-MDA5^+^ DM patients in order to investigate the role of different anti-MDA5^+^ phenotypes in predicting outcomes. The identification of three distinct clinical patterns and outcomes ultimately suggested that the classification of these patients into phenotypes with prognostic value could aid clinicians in making effective clinical decisions. A study by Sugita T. et al. (36) demonstrated that the presence of the anti-Ro-52 antibody is frequently observed in patients diagnosed with DM/PM, suggesting its potential as a diagnostic marker for disease severity. In the aforementioned study, the incidence of glucocorticoid-resistant cardiac involvement was found to be significantly higher in the anti-Ro-52 antibody-positive group. As demonstrated in (42), the anti-Ro-52 antibody has been associated with an elevated risk of cardiac injury. To date, no study has directly established a link between the anti-Ro-52 antibody and cardiac involvement in DM/PM, and its specificity is relatively low. Consequently, the anti-Ro-52 antibody may function as an auxiliary reference indicator for cardiac involvement in DM/PM.

Despite the fact that the direct association between MSAs, MAAs, and specific cardiac pathologies remains an area of active investigation, the detection of antibody profiles aids in identifying patient populations at risk for specific complications, thereby enabling the implementation of more proactive cardiac evaluation and early intervention (Figure 1).

## 4 Multimodal imaging evaluation of cardiac involvement in DM/PM

### 4.1 Echocardiography

Echocardiography is the primary screening tool for assessing cardiac structure and function. The advantages of this procedure are that it is non-invasive, convenient, repeatable, and capable of real-time imaging. Tissue Doppler Imaging (TDI), a technology based on echocardiography, has been demonstrated to be capable of sensitively capturing motion signals of myocardial tissue. However, there is currently a paucity of research on TDI for measuring left ventricular diastolic dysfunction in DM/PM. The extant literature suggests that the likelihood of left ventricular diastolic dysfunction in patients with DM (76.5%) is considerably higher than that observed in the control group (52.9%) (43). In a study by Péter A et al. (44), conventional echocardiographic parameters (systolic and diastolic) were measured in 30 patients with DM/PM who did not exhibit cardiovascular symptoms. The study concluded that conventional echocardiography combined with TDI has high sensitivity for identifying subclinical changes in myocardial structure and function. TDI has been shown to serve as a supplement to conventional echocardiographic measurements, offering a rapid means of identifying early cardiac signs in patients with DM/PM.

Speckle-tracking echocardiography (STE) is a novel, non-invasive echocardiographic technique that can assess subclinical myocardial involvement through the measurement of global longitudinal strain (GLS) (45). Guerra F et al. (46) utilized STE to evaluate GLS in patients with DM/PM, thereby identifying a higher prevalence of subclinical systolic dysfunction in both the left and right ventricles. In the study by Zhong Y et al. (47), three-dimensional speckle tracking echocardiography (3D-STE) was employed, thus demonstrating that DM/PM patients with a normal left ventricular ejection fraction (LVEF) exhibited significantly reduced left ventricular GLS and right ventricular GLS in comparison to healthy controls (all *P* < 0.001). Consequently, STE technology has the capacity to provide crucial evidence for the early identification of subclinical cardiac involvement in DM/PM, and GLS may serve as an important indicator for risk stratification of cardiac involvement.

Myocardial work (MW) is a novel non-invasive technique for assessing left ventricular systolic function. This technology integrates left ventricular GLS measured by STE, with left ventricular pressure estimated non-invasively via brachial artery cuff pressure to construct a left ventricular pressure-strain loop (PSL) (48). This methodology enables the quantitative derivation of a series of myocardial work parameters, including the global work index (GWI), global constructive work (GCW), global wasted work (GWW), and global work efficiency (GWE). At present, this technique has been applied in various fields, including coronary artery disease, hypertrophic cardiomyopathy, and myocardial injury related to pulmonary hypertension (49–51). Patients with DM/PM frequently have concomitant interstitial lung disease (52) and a high risk of pulmonary hypertension, which can easily lead to alterations in afterload. MW has been shown to overcome the limitations of conventional echocardiography and STE, which are both susceptible to afterload influence. This enables a more authentic and objective reflection of myocardial contractile function and energy expenditure. However, to date, no relevant studies on the application of MW technology for evaluating cardiac involvement in patients with DM/PM have been retrieved. MW has been shown to possess the capability to sensitively identify subclinical myocardial functional impairment while avoiding the confounding effect of afterload, thus holding significant promise for playing an important role in the future assessment of cardiac function in patients with DM/PM (Figure 2).

**Figure 2:**
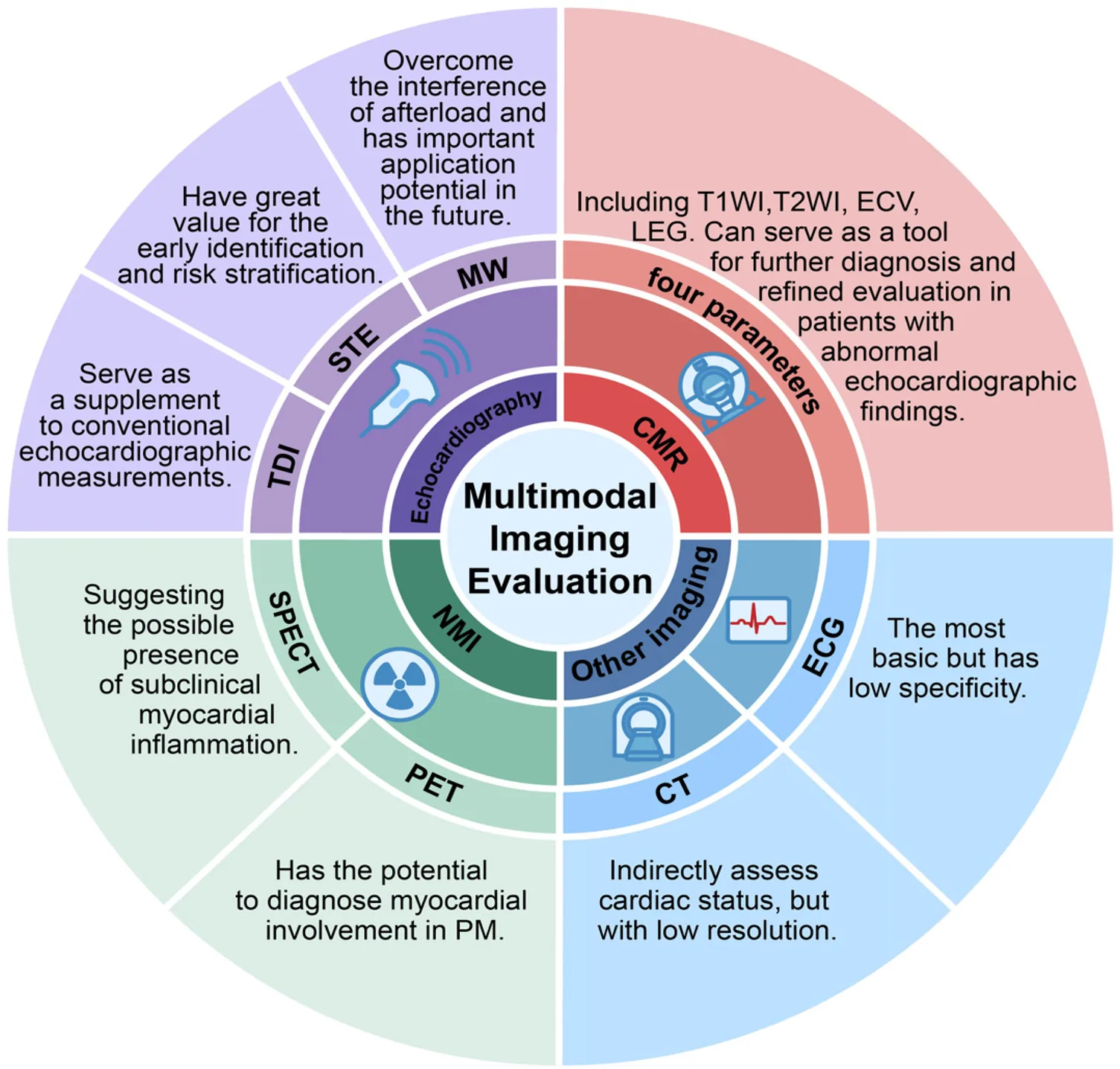
Multimodal imaging evaluation of cardiac involvement in DM/PM. The schematic illustrates complementary roles of echocardiography (including STE and TDI), cardiac magnetic resonance (CMR with four parameters), ECG, CT, PET, and SPECT in assessing myocardial damage, inflammation, fibrosis, and perfusion.

### 4.2 Cardiac magnetic resonance imaging (CMR)

CMR is characterized by its high temporal and spatial resolution, which facilitates the early detection of myocardial pathologies, including fibrosis, oedema, and inflammatory infiltration. The imaging parameters that are most frequently employed in such cases include T1-weighted imaging, T2-weighted imaging, quantification of extracellular volume (ECV), and late gadolinium enhancement (LGE) imaging. It is widely regarded as the gold standard for the non-invasive quantification of ventricular volumes and ejection fraction (53). A retrospective study by Feng C. et al. (54) analyzed CMR parameters in 44 DM/PM patients with normal left ventricular morphology and function. The study observed elevated global native T1 and ECV values, then concluded that CMR tissue characterization can detect early myocardial involvement in DM/PM patients without overt left ventricular dysfunction. This allows for the monitoring of early cardiac involvement, timely clinical warning, and implementation of therapeutic measures. Nevertheless, CMR examination is associated with higher costs and longer scan times, and is contraindicated in patients with certain metallic implants. In clinical practice, its utilization should be judiciously selected based on the unique circumstances of each patient. It can serve as a further diagnostic and detailed assessment tool for patients with abnormal echocardiographic findings (Figure 2).

### 4.3 Nuclear medicine imaging

Nuclear medicine imaging primarily relies on the use of radionuclide-labeled imaging agents to reflect myocardial metabolism, perfusion, and inflammation. At present, the clinical nuclear imaging techniques most frequently employed are Single Photon Emission Computed Tomography (SPECT) and Positron Emission Tomography (PET). The SPECT agent that is most frequently used is ⁹⁹ᵐTc. A study by Diederichsen LP et al. (55) indicated that semi-quantitative ⁹⁹ᵐTechnetium-pyrophosphate (⁹⁹ᵐTc-PYP) scintigraphy is an advanced and sensitive technique for the non-invasive detection of myocardial inflammation. Despite the absence of a statistically significant disparity in myocardial ⁹⁹ᵐTc-PYP uptake between the patient cohort and the healthy control group, a notable correlation was identified between high myocardial ⁹⁹ᵐTc-PYP uptake and left ventricular diastolic dysfunction within the experimental group. The presence of elevated ⁹⁹ᵐTc-PYP uptake is indicative of inflammation, which may be subclinical, with concomitant preserved systolic function. The PET agent most frequently employed is Fluorodeoxyglucose (FDG). FDG-PET has been demonstrated to be a valuable tool in the diagnosis of inflammatory lesions in skeletal muscle, with the potential to detect various inflammatory cardiovascular diseases, including PM (56). It may serve as an ancillary instrument to facilitate the diagnosis of myocarditis in patients with DM/PM (57). Nuclear medicine imaging is a suitable modality for the early screening and assessment of the extent of cardiac involvement in DM/PM, particularly for patients unable to undergo CMR examination. However, it frequently necessitates combination with other imaging techniques and biomarkers for differential diagnosis and can serve as a supplementary means for early warning and risk assessment of cardiac involvement (Figure 2).

### 4.4 Other imaging and auxiliary examinations

Electrocardiography (ECG) is widely regarded as the most fundamental clinical cardiac examination. It is a rapid screening procedure that can detect abnormalities, including arrhythmias and myocardial ischemia. A study by Schwartz T et al. (58) found that among 58 JDM patients, 17% exhibited ECG abnormalities, a prevalence significantly higher than that in the control group (*P* = 0.02). Although cardiac dysfunction in such patients often remains in a subclinical state, this may indicate an increased risk of developing overt clinical heart disease in the future. However, ECG has low specificity, and it can be normal in many patients with subclinical cardiac involvement. Therefore, its findings require comprehensive interpretation in conjunction with other diagnostic modalities. Chest computed tomography (CT) is primarily utilized to assess pulmonary manifestations in patients with DM/PM, including interstitial lung disease (59). Furthermore, it can also indirectly assess cardiac dimensions, morphology, and pericardial conditions, detecting abnormalities such as left ventricular enlargement, pericardial thickening, or pericardial effusion. However, its resolution for cardiac structures is inferior to that of echocardiography and CMR, thus rendering it merely an auxiliary assessment tool (Figure 2).

## 5 Clinical application: risk assessment system for DM/PM cardiac involvement

The risk assessment system for cardiac involvement in DM/PM can be constructed through the integration of multidimensional information, including biomarkers, multimodal imaging evaluation, clinical features, and associated risk factors. The system under discussion possesses the advantages of being multidimensional and individualized, thereby enabling stratified management of patients, enhanced monitoring and early intervention, and ultimately improved prognosis. At present, a unified clinical standard for risk assessment of cardiac involvement in DM/PM does not exist. In light of recent advancements in the field, the process can be established as a synergistic three-stage model encompassing early-warning screening, stratified evaluation, and prognostic assessment.

Although endomyocardial biopsy remains the pathological gold standard for myocarditis, it is not routinely performed in IIM-associated cardiac involvement because of its invasive nature, limited sensitivity due to sampling error, and procedural risk. However, EMB may be considered in selected patients with unexplained new-onset heart failure, refractory or fulminant arrhythmias, rapidly progressive cardiomyopathy, or suspected myocarditis when noninvasive assessment is inconclusive and histological confirmation would influence therapeutic decision-making, particularly the initiation or escalation of immunosuppressive treatment or the exclusion of alternative etiologies such as infiltrative, infectious, or toxic cardiomyopathy.

### 5.1 Early-warning screening for patient populations

Early-warning screening can be achieved through a number of methods, including basic clinical evaluation and physical examination, traditional myocardial injury markers and inflammatory markers, autoantibodies, and conventional echocardiography. For patients who have been newly diagnosed or are undergoing follow-up with a diagnosis of DM/PM, it is recommended that routine screening be performed for cTnI, CK, inflammatory markers, and the myositis-specific autoantibody profile. For individuals exhibiting normal results, the necessity of regular follow-up appointments remains paramount. Should the results be deemed to be abnormal, and thus indicating a potential risk of cardiac involvement, further investigations should be conducted. Such further investigations may include ECG and echocardiography. In the event of persistent abnormalities, a stratified assessment, utilizing modalities such as CMR, may be indicated, with this decision being made based on the prevailing clinical context. This process facilitates the identification of high-risk individuals from a large patient population, thereby enabling the early detection of cardiac involvement.

### 5.2 Antibody subtype-guided stratified evaluation and prognostic assessment

Certain autoantibody profiles may help identify patient subgroups requiring closer cardiac surveillance; however, current evidence does not support a validated antibody-based risk stratification system for cardiac involvement in DM/PM. Autoantibodies should therefore be interpreted as supportive clinical risk indicators rather than standalone tools for cardiac risk grading. In the case of DM/PM patients with confirmed cardiac involvement, improvements in cardiac function can still be evaluated by changes in biomarker levels and multimodal imaging findings. Should post-treatment biomarker levels, such as cTn and CK, demonstrate a downward trend in conjunction with the observed alleviation in imaging studies, this would indicate treatment efficacy, and the current therapeutic regimen can be maintained with continued regular follow-up. Conversely, if biomarker levels show persistent increase and imaging reveals disease progression, this indicates a poor prognosis, necessitating timely adjustment of the treatment plan and intensification of cardiac interventions. However, this strategy should be regarded as a proposed framework, as direct prospective validation in DM/PM is still lacking.

In addition, anti-SRP antibody positivity deserves special attention. Anti-SRP is characteristically associated with IMNM, a contemporary IIM subtype that is increasingly recognized as distinct from historically defined PM. Anti-SRP-positive patients may exhibit a higher prevalence of cardiac involvement, including myocardial injury, arrhythmia, and ventricular dysfunction. Therefore, anti-SRP positivity should be considered a high-risk feature prompting closer cardiac evaluation and follow-up. This is particularly relevant because some patients historically classified as PM may, under current classification systems, actually belong to the IMNM spectrum.

### 5.3 Application in special populations

Special populations deserve particular consideration in cardiac risk stratification across the IIM spectrum.

First, risk may differ substantially among IIM subgroups. For example, anti-SRP-positive patients, typically classified within IMNM, appear to be at increased risk of cardiac involvement and may require more intensive screening. Second, traditional cardiovascular risk factors, including older age, hypertension, diabetes mellitus, dyslipidemia, obesity, and smoking, may amplify overall cardiovascular risk and complicate the interpretation of elevated cardiac biomarkers or imaging abnormalities. In these patients, a multimodal evaluation integrating IIM subtype, autoantibody profile, disease activity, treatment exposure, and conventional cardiovascular risk burden may provide a more accurate assessment than any single parameter alone.

As indicated by the existing literature (60), JDM shares similarities with adult-onset DM regard to pathological mechanisms and clinical manifestations. However, challenges such as inaccurate symptom description and poor cooperation are more prevalent in children. Consequently, for pediatric patients with DM/PM, priority should be given to screening based on more objective biomarkers, such as highly specific cTnI combined with echocardiography. Conversely, elderly DM/PM patients exhibit an increased vulnerability to cardiovascular risk factors or established cardiovascular diseases, attributable to the natural progression of age-related conditions. This renders the early warning and assessment of cardiac involvement a more intricate process. Consequently, a combined strategy is necessary. This includes the utilization of high-specificity markers such as cTnI to monitor myocardial injury, the employment of inflammatory markers to reflect the level of systemic inflammation, and the reliance on MSAs to indicate specific risk subtypes. It is imperative that this approach is integrated with ECG, echocardiography, and CMR. When indicated, coronary computed tomography angiography or conventional angiography can be employed to assist in determining the extent of cardiac involvement. Furthermore, it is recommended that this population be subject to more intensive cardiac follow-up.

## 6 Conclusions and future outlook

Cardiac involvement in DM/PM frequently manifests with an insidious onset, which can be easily overlooked in clinical practice. This underscores the importance of early recognition and precise assessment for improving prognosis. Biomarkers have been shown to provide serological clues that reflect the extent of myocardial inflammation and injury. The combination of traditional and emerging biomarkers has the potential to enhance the accuracy of early warning systems. Multimodal imaging techniques have been developed to facilitate direct visualization of structural cardiac alterations. Complementary tools, including echocardiography as a routine screening method, CMR for precise evaluation of myocardial pathology, and nuclear medicine imaging, enable a comprehensive assessment of cardiac involvement. The integration of biomarkers with multimodal imaging and clinical high-risk factors forms a multidimensional, individualized risk assessment system. The system has the capacity to facilitate early warning screening, stratified evaluation, and prognosis assessment for cardiac involvement in DM/PM patients, thereby guiding personalized clinical intervention. We note that high-quality DM/PM-specific studies published in the last 5 years remain limited in several domains. Therefore, some earlier studies were retained because they continue to represent the core evidence base for this topic. At present, evidence supporting novel biomarkers, integrated multimodal imaging pathways, and formal cardiac risk stratification in DM/PM remains limited, and further prospective DM/PM-specific studies are needed.

It is recommended that future research efforts concentrate on the establishment of large-scale, prospective cohort studies. The primary objective of such studies would be to validate the clinical utility of various biomarkers. A secondary objective would be to standardize their cut-off values. The development of multi-biomarker combined prediction models is also recommended, with a view to enhancing the sensitivity and specificity of early warning for cardiac involvement in DM/PM. Concurrently, the integration of imaging technologies with artificial intelligence (AI) has the potential to facilitate automated image analysis and lesion quantification, thereby improving the convenience and accuracy of detection and assessment. Despite the current state of research, which is characterized by numerous shortcomings, the continuous advancement of biomarker detection and multimodal imaging technologies, as well as deeper insights into the underlying mechanisms, will contribute to the enhancement of the ability to provide early warning and risk assessment for cardiac involvement in DM/PM. This progress offers the potential for early detection and timely intervention, which may ultimately delay disease progression and improve the long-term prognosis of patients (Figure 3).

**Figure 3:**
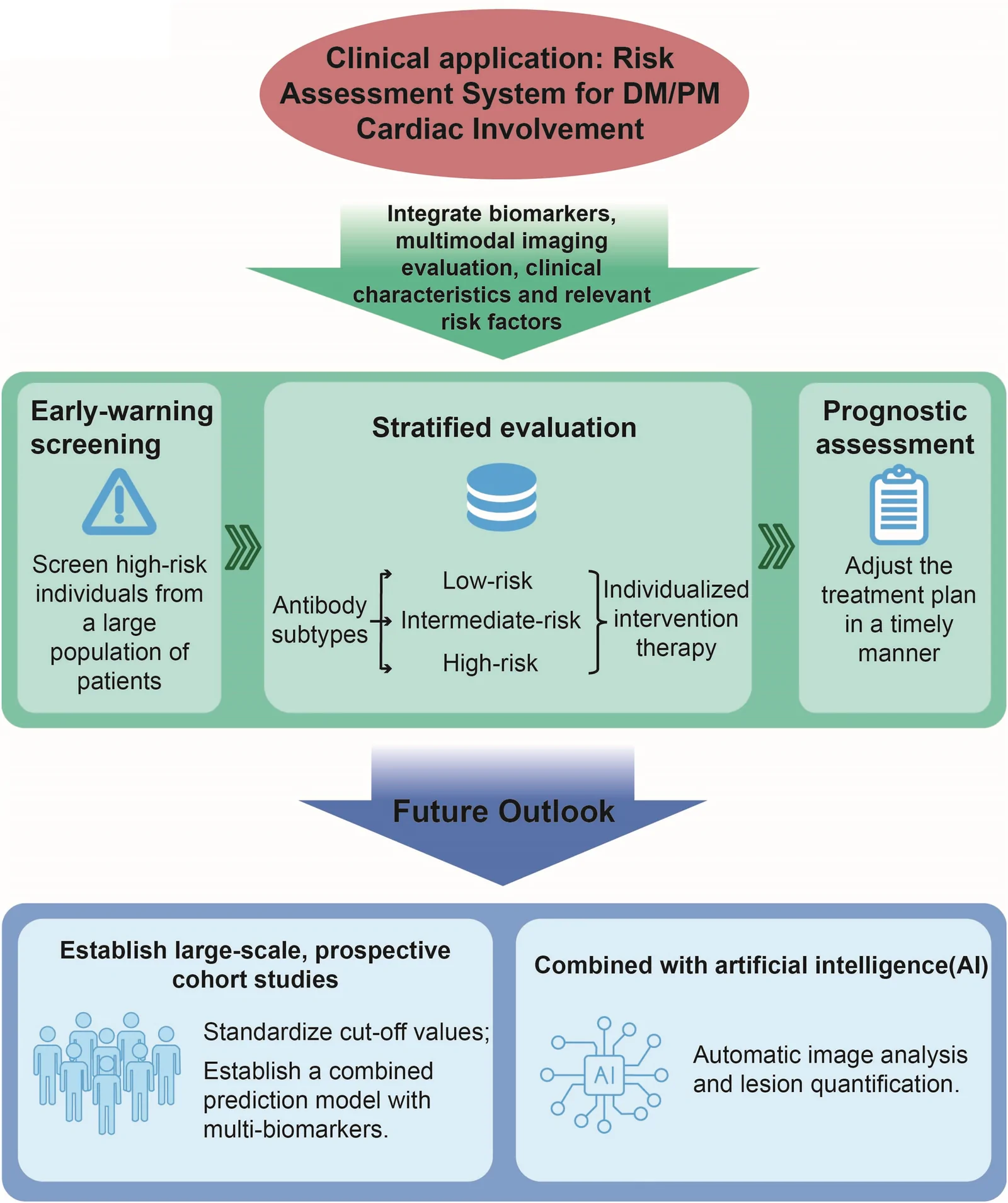
Clinical application of the risk assessment system for cardiac involvement in DM/PM and future outlook. The system integrates biomarkers, multimodal imaging, clinical features, and risk factors to enable early-warning screening of high-risk individuals from large patient cohorts, guide individualized intervention therapy and assess prognostic to timely adjust treatment plans. Future outlook includes establishing large-scale prospective cohort studies to standardize cut-off values and develop multi-biomarker prediction models, as well as integrating artificial intelligence (AI) for automated image analysis and lesion quantification.
